# Genetic determinants of clinical phenotype in hypertrophic cardiomyopathy

**DOI:** 10.1186/s12872-020-01807-4

**Published:** 2020-12-09

**Authors:** Lazar Velicki, Djordje G. Jakovljevic, Andrej Preveden, Miodrag Golubovic, Marija Bjelobrk, Aleksandra Ilic, Snezana Stojsic, Fausto Barlocco, Maria Tafelmeier, Nduka Okwose, Milorad Tesic, Paul Brennan, Dejana Popovic, Arsen Ristic, Guy A. MacGowan, Nenad Filipovic, Lars S. Maier, Iacopo Olivotto

**Affiliations:** 1 Faculty of Medicine, University of Novi Sad, Novi Sad, Serbia; 2 Institute of Cardiovascular Diseases of Vojvodina, Sremska Kamenica, Serbia; 3 Cardiovascular Research, Translational and Clinical Research Institute, Medicine, Newcastle University, Newcastle upon Tyne Hospitals NHS Foundation Trust, Newcastle upon Tyne, UK; 4 Careggi University Hospital, University of Florence, Florence, Italy; 5 Department of Internal Medicine II (Cardiology, Pneumology, and Intensive Care), University Medical Centre Regensburg, Regensburg, Germany; 6 Cardiology Department, Clinical Centre of Serbia, Faculties of Medicine and Pharmacy, University of Belgrade, Belgrade, Serbia; 7 Bioengineering Research and Development Center, BioIRC, Kragujevac, Serbia; 8 Faculty of Engineering, University of Kragujevac, Kragujevac, Serbia; 9 Faculty of Health and Life Sciences, Coventry University, and University Hospitals Coventry and Warwickshire NHS Trust, Coventry, UK

**Keywords:** Hypertrophic cardiomyopathy, HCM, Hereditary cardiac disease, Left ventricular hypertrophy, MYBPC3, MYH7

## Abstract

**Background:**

Hypertrophic cardiomyopathy (HCM) is the most common inherited cardiovascular disease that affects approximately one in 500 people. HCM is a recognized genetic disorder most often caused by mutations involving myosin-binding protein C (MYBPC3) and β-myosin heavy chain (MYH7) which are responsible for approximately three-quarters of the identified mutations.

**Methods:**

As a part of the international multidisciplinary SILICOFCM project (www.silicofcm.eu) the present study evaluated the association between underlying genetic mutations and clinical phenotype in patients with HCM. Only patients with confirmed single pathogenic mutations in either MYBPC3 or MYH7 genes were included in the study and divided into two groups accordingly. The MYBPC3 group was comprised of 48 patients (76%), while the MYH7 group included 15 patients (24%). Each patient underwent clinical examination and echocardiography.

**Results:**

The most prevalent symptom in patients with MYBPC3 was dyspnea (44%), whereas in patients with MYH7 it was palpitations (33%). The MYBPC3 group had a significantly higher number of patients with a positive family history of HCM (46% vs. 7%; *p* = 0.014). There was a numerically higher prevalence of atrial fibrillation in the MYH7 group (60% vs. 35%, *p* = 0.085). Laboratory analyses revealed normal levels of creatinine (85.5 ± 18.3 vs. 81.3 ± 16.4 µmol/l; *p* = 0.487) and blood urea nitrogen (10.2 ± 15.6 vs. 6.9 ± 3.9 mmol/l; *p* = 0.472) which were similar in both groups. The systolic anterior motion presence was significantly more frequent in patients carrying MYH7 mutation (33% vs. 10%; *p* = 0.025), as well as mitral leaflet abnormalities (40% vs. 19%; *p* = 0.039). Calcifications of mitral annulus were registered only in MYH7 patients (20% vs. 0%; *p* = 0.001). The difference in diastolic function, i.e. E/e′ ratio between the two groups was also noted (MYBPC3 8.8 ± 3.3, MYH7 13.9 ± 6.9, *p* = 0.079).

**Conclusions:**

Major findings of the present study corroborate the notion that MYH7 gene mutation patients are presented with more pronounced disease severity than those with MYBPC3.

## Background

Hypertrophic cardiomyopathy (HCM) is the most frequent inherited disease of the myocardium, with a prevalence of approximately 0.2% [1, 2]. Despite the significant developments in diagnostic tools and genetic tests, the diagnosis of HCM is often delayed [2].

HCM is characterized by left ventricular (LV) hypertrophy without dilatation, in the absence of any other cardiac, systemic, metabolic, or syndromic disease that could explain myocardial hypertrophy [2–5]. Clinical presentation of HCM varies from completely asymptomatic with normal life expectancy, to typical symptoms like chest pain, shortness of breath, heart failure, palpitations, syncope, and in the worst case even sudden cardiac death [2, 6]. Complications of non-obstructive HCM include advanced myocardial fibrosis, microvascular ischemia, and deterioration of cardiac function [7].

HCM is a recognized genetic disorder transmitted in an autosomal dominant fashion, caused by a single mutation in one of the sarcomeric protein genes, which can be present in either thick- or thin-filament genes [8, 9]. The two most common mutations involving thick filament are myosin-binding protein C (MYBPC3) and β-myosin heavy chain (MYH7) gene mutations, which are responsible for approximately three-quarters of the identified mutations in HCM patients [9, 10]. Aside from these two, a few other less frequent gene mutations (e.g. troponin I type 3 [TNNI3], troponin T type 2 [TNNT2], α-tropomyosin [TPM1], α-actin [ACTC]) are possible causes of HCM as well and are therefore also included in the routine HCM genetic testing [11]. Technological progress has made it possible to identify new genes associated with HCM—numerous other genes that do not encode sarcomere proteins but rather genes encoding the synthesis of Z-disk proteins and proteins involved in the calcium signaling pathway. With the introduction and implementation of the next-generation sequencing solutions, the identification of nearly 50 gene mutations associated with some form of HCM throughout literature has become possible [12].

Regardless of the mutation type, the same pathophysiology mechanisms are responsible for the development of typical HCM phenotype and disease progression. Disrupted sarcomere properties due to the mutations cause impaired relaxation and lead to diastolic dysfunction, which is followed by hyperdynamic contractility and hypertrophy of the LV in the later course [9, 11].

Due to variable penetrance and expressivity, the phenotypic characteristics of HCM are multifaceted and may be influenced by other factors beyond single pathogenic mutations [13]. In addition to LV hypertrophy, phenotypic HCM expression also includes myocardial hypercontractivity, myofibril disorganization, fibrosis, as well as the presence of mild myocardial inflammation. Although the clinical phenotype can partially differ depending on the affected gene, no distinctive correlation between disease severity and specific genes has been established. Moreover, clinical features such as disease penetration, hypertrophy severity, and patient prognosis are known to vary depending on different mutations within the same gene [11].

The precise link between determined underlying gene mutation and the clinical course remains elusive in this heterogeneous condition. The motivation to compile this HCM patient registry was to try to define what patient features are more prevalent with specific gene mutations and to establish whether the level of disease expression might be linked to one of the two most common mutations responsible for HCM. The goal was to reveal and distinguish subtle differences that may exist in clinical presentation and, more importantly, in heart structure and function recorded by cardiac imaging (i.e. echocardiography) between different gene mutations, thus providing essential information for the computational model development. Moreover, data from this study will also complement the clinical trial (NCT03832660 at *clinicaltrials.gov*) evaluating the effects of pharmacological (sacubitril/valsartan) versus lifestyle intervention in HCM patients [14], also a fundamental part of the SILICOFCM project.

## Methods

As a part of the international multidisciplinary SILICOFCM project (www.silicofcm.eu) developing a computational platform for in silico clinical trials of familial cardiomyopathies, the present study evaluated the association between genetic mutations and clinical phenotype in patients with HCM. The study protocol was approved by the UK National Health Service Health Research Authority North East—Tyne & Wear South Research Ethics Committee with the reference number 18/NE/0318 and was adopted by the Institutional Review Board of each participating center. All patients provided written informed consent and all procedures were conducted following the Declaration of Helsinki.

### Study design

Participating centers included patients with diagnoses of HCM who were identified in the period from June 2018 to February 2019.

The diagnosis of HCM was defined according to the European Society of Cardiology guidelines i.e. maximal LV wall thickness of ≥ 15 mm on echocardiography, in the absence of any other cardiac or systemic disease that would be capable of producing myocardial hypertrophy, such as afterload abnormalities like aortic valve stenosis or arterial hypertension [5]. Of the total of 74 HCM patients, 11 were excluded because of the relatively small number of other gene mutations (TNNI3—5 patients, TNNT2—2 patients, TPM1—1 patient, myosin heavy chain 6—1 patient, myosin light chain 2—1 patient, lamin A/C—1 patient) might have biased the overall study analysis. In the final analysis, the study included a total of 63 adult patients with a confirmed diagnosis of HCM.

We excluded patients with significant atherosclerotic coronary artery disease (> 50% stenosis in a major artery), patients with prior cardiac surgery (including septal myectomy), alcohol septal ablation, major LV outflow obstruction with pressure gradient > 50 mmHg, and chronic renal failure (< 30 ml/min/1.73 m^2^).

### Genetic testing

Genetic testing was performed from peripheral blood samples acquired by phlebotomy with the utilization of the QIAamp DNA Blood BioRobot MDx kit (QIAGEN GmbH, Hilden, Germany). Polymerase chain reaction with primers was used for the amplification of candidate exons.

Blood samples were analyzed for the presence of the 8 most common mutations, which represent the basis of the commonly available genetic tests for HCM. These mutations include the protein-coding exons responsible for encoding myosin-binding protein C (MYBPC3), thick-filament proteins (β-myosin heavy chain [MYH7] and the regulatory and essential light chains [MYL2 and MYL3]), and thin-filament proteins (troponin T type 2 [TNNT2], troponin I type 3 [TNNI3], α-tropomyosin [TPM1], and α-actin [ACTC]).

Only patients with confirmed single pathogenic mutations in either MYBPC3 or MYH7 genes were included in the study. Based on the identified gene mutation the patients were divided into two groups. The MYBPC3 group was comprised of 48 patients (76%), while the MYH7 group included 15 patients (24%).

### Electrocardiogram and ECG Holter monitoring

The ECG was performed using a standard 12-lead­electrocardiogram in a supine position. To identify sporadic arrhythmia, all participants were asked to wear an ECG-Holter monitor for 24 h and to keep a diary of activities and symptoms.

### Echocardiography

Transthoracic echocardiography was performed in all patients. Images were obtained using regular parasternal and apical views. All the parameters were calculated and indexed for body surface area (BSA).

LV wall thickness and chamber dimensions were measured using the parasternal long-axis view [14, 15]. The Devereux formula [16] was used to calculate LV myocardial mass.

LV geometry was assessed by the relative wall thickness which is calculated as two times the LV posterior wall thickness divided by LV end-diastolic diameter.

LV systolic and diastolic volumes were measured with Simpson’s modified biplane method using apical 4-chamber and 2-chamber views, and LV systolic function was expressed through the ejection fraction [15].

For diastolic function assessment, an apical 4-chamber view was used [17]. Blood flow through the mitral valve was measured by pulsed-wave Doppler between the tips of mitral leaflets and the peak modal velocity in early diastole (E) was determined. Velocities of basal regions at lateral and septal mitral annulus were recorded using tissue Doppler imaging and then their average ratio (e′) was computed.

The filling pressure of the LV was expressed through the E/e′ ratio, which is the most accurate indicator of diastolic function according to the literature [18].

### Statistical analysis

Continuous variables are expressed as mean values ± standard deviation and categorical variables are presented as absolute numbers and percentages. Quantitative data distribution was assessed using the Kolmogorov–Smirnov test. Mean values of continuous variables were compared using the independent samples t-test or Mann–Whitney U test, whereas categorical variables were compared using the chi-square test. Statistical significance for all tests was set at the *p* value of < 0.05. All the analyses were done in SPSS version 20.0.

## Results

The mean age of HCM patients regardless of genetic mutation was 51.1 ± 14.2 years and most of them were male 48 (76%). They were slightly overweight according to their mean BMI of 26.4 ± 4.4 kg/m^2^. One-third of patients (36%) had a positive family history for HCM.

Differences in terms of patient profile depending on genetic mutation are shown in Table 1.

**Table 1:** General characteristics of patients with MYBPC3 and MYH7 gene mutation

	Overall	MYBPC3	MYH7	*p* value
Age (years)	51.1 ± 14.2	49.8 ± 14.3	55.1 ± 13.3	0.211
Females, *n* (%)	15 (23.8%)	10 (20.8%)	5 (33.3%)	0.321
BMI (kg/m^2^)	26.4 ± 4.4	26.1 ± 4.6	27.8 ± 3.1	0.260
Fatigue, *n* (%)	9 (14.3%)	7 (14.6%)	2 (13.3%)	0.881
Dyspnea, *n* (%)	25 (39.7%)	21 (43.7%)	4 (26.7%)	0.238
Chest pain, *n* (%)	6 (9.5%)	4 (8.3%)	2 (13.3%)	0.565
Palpitations, *n* (%)	13 (20.6%)	8 (16.7%)	5 (33.3%)	0.177
Syncope, *n* (%)	10 (15.9%)	9 (18.7%)	1 (6.6%)	0.264
Family history of HCM, *n* (%)	23 (36.5%)	22 (45.8%)	1 (6.6%)	0.014*
Comorbidities				
Diabetes mellitus, *n* (%)	3 (4.8%)	3 (6.2%)	–	–
Chronic obstructive pulmonary disease, *n* (%)	2 (3.2%)	2 (4.2%)	–	–
Thyroid dysfunction, *n* (%)	8 (12.7%)	7 (14.6%)	1 (6.7%)	0.422
Anemia, *n* (%)	1 (1.6%)	1 (2.1%)	–	–
Laboratory analyses				
Glucose (mmol/l)	5.6 ± 1.2	5.8 ± 1.3	5.0 ± 0.6	0.071
Creatinine (µmol/l)	84.4 ± 17.7	85.5 ± 18.3	81.3 ± 16.4	0.487
Blood urea nitrogen (mmol/l)	9.0 ± 12.7	10.2 ± 15.6	6.9 ± 3.9	0.472
ALT (U/l)	30.1 ± 15.7	31.8 ± 17.0	25.0 ± 10.4	0.268
Total protein (g/l)	69.1 ± 8.4	69.3 ± 7.2	68.6 ± 11.1	0.853
Albumin (g/l)	44.0 ± 6.8	44.1 ± 6.4	43.9 ± 8.0	0.948
Sodium (mmol/l)	140.3 ± 2.1	140.4 ± 2.1	140.2 ± 2.3	0.868
Potassium (mmol/l)	4.5 ± 0.4	4.5 ± 0.3	4.6 ± 0.5	0.531
Calcium (mmol/l)	2.3 ± 0.1	2.3 ± 0.1	2.3 ± 0.2	0.689
NT-proBNP (ng/l)	1328.3 ± 1420.2	1304.5 ± 1457.5	1757.2 ± 1335.2	0.766

*ALT* alanine transaminase, *BMI* body mass index, *HCM* hypertrophic cardiomyopathy, *NT-proBNP* N-terminal pro-brain natriuretic peptide

There was no significant difference between patients carrying the MYBPC3 and MYH7 mutations regarding age (49.8 ± 14.3 vs. 55.1 ± 13.3 years, *p* = 0.211) and gender distribution (21% vs. 33% females, *p* = 0.321).

The most prevalent symptom in patients with MYBPC3 was dyspnea (44%), whereas in patients with MYH7 it was palpitations (33%). Other less frequently reported symptoms included fatigue, chest pain, and syncope, with similar distribution among the groups.

Interestingly, the MYBPC3 group had a significantly higher number of patients with a positive family history of HCM (46% vs. 7%; *p* = 0.014).

The most frequently found comorbidity was thyroid gland dysfunction, which was present in 8 patients (13%) in total, without significant difference between MYBPC3 and MYH7 groups (15% vs. 7%; *p* = 0.422). No significant difference between the MYBPC3 and MYH7 patients was observed in other comorbidities as well: diabetes mellitus (6% vs. 0%; *p* = 0.321), chronic obstructive pulmonary disease (4% vs. 0%; *p* = 0.422), anemia (2% vs. 0%; *p* = 0.573).

The mean heart rate was similar between MYBPC3 and MYH7 patients (64.6 ± 11.8 vs. 67.8 ± 20.4 bpm; *p* = 0.546). However, there was a numerically higher prevalence of atrial fibrillation in the MYH7 group (60% vs. 35%, *p* = 0.085).

Blood laboratory analyses indicating renal and liver function, as well as blood glucose and electrolytes, showed levels within the reference range, without differences between MYBPC3 and MYH7 patients (Table 1). Levels of N-terminal pro-brain natriuretic peptide (NT-proBNP) were elevated in all patients, but without difference among groups.

**Fig. 1:**
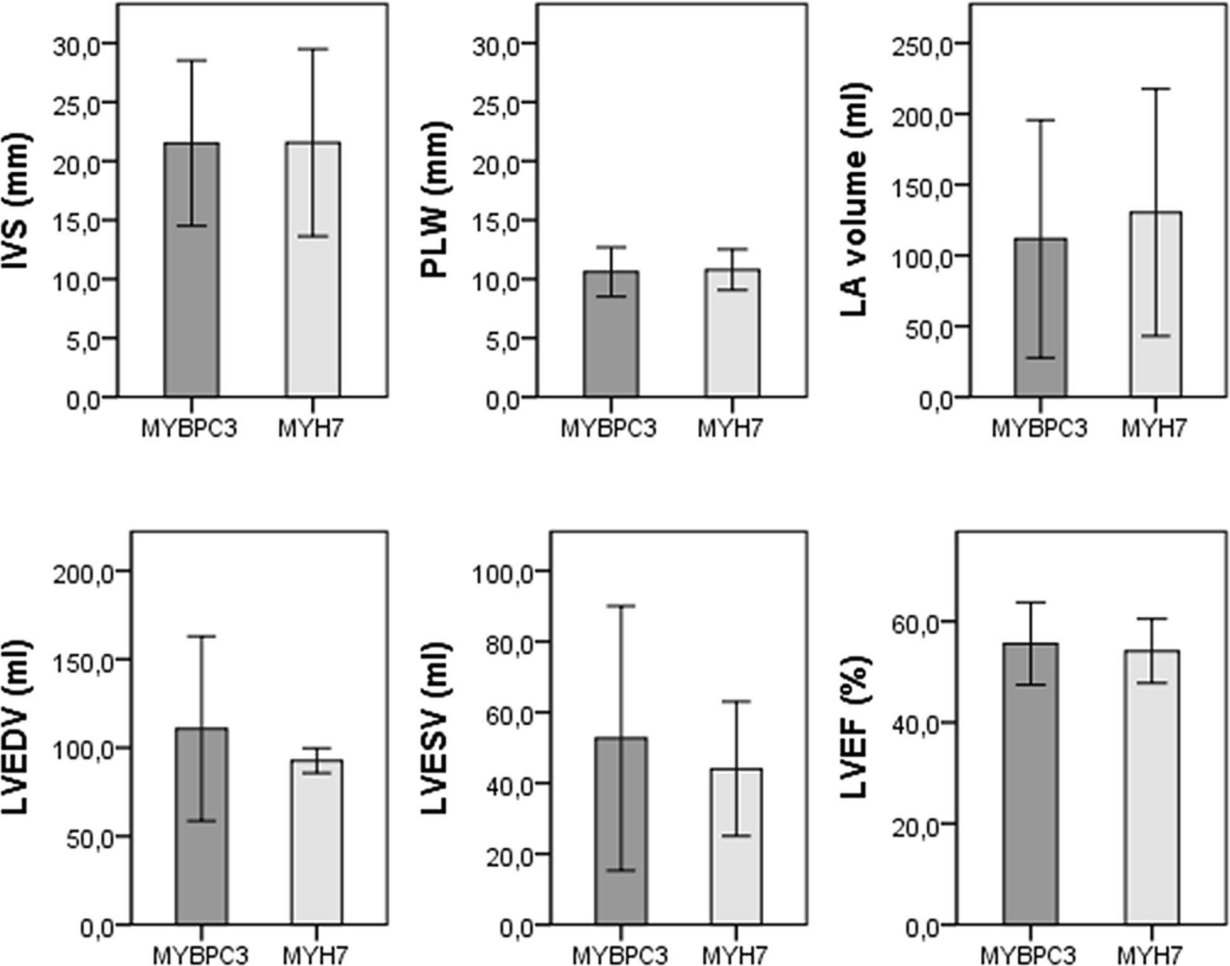
Echocardiography parameters in MYBPC3 and MYH7 patients (no significant difference was observed in the presented parameters, *p* > 0.05) (*IVS* interventricular septum, *PLW* posterolateral wall, *LA* left atrium, *LVEDV* left ventricle end-diastolic volume, *LVESV* left ventricle end-systolic volume, *LVEF* left ventricle ejection fraction)

Echocardiography findings are presented in Table 2 and Fig. 1. There was no difference in the posterolateral wall (10.6 ± 2.1 vs. 10.8 ± 1.7 mm, *p* = 0.776) and interventricular septum (21.5 ± 7.0 vs. 21.6 ± 7.9 mm, *p* = 0.982) thickness between MYBPC3 and MYH7 patients. Left atrial volume was 14% lower (*p* = 0.518) and left ventricular end-diastolic volume was 19% higher (*p* = 0.560) in MYBPC3. Left ventricular end-systolic volume was similar between MYBPC3 and MYH7 (52.6 ± 37.4 vs. 44.0 ± 19.0 ml; *p* = 0.700) as was left ventricular ejection fraction (55.6 ± 8.2 vs. 54.1 ± 6.3, *p* = 0.594) and tricuspid annular plane systolic excursion (TAPSE) (21.0 ± 4.4 vs. 22.5 ± 6.0 mm, *p* = 0.363).

**Table 2:** Echocardiography findings in patients with MYBPC3 and MYH7 gene mutation

	Overall	MYBPC3	MYH7	*p* value
PLW thickness (mm)	10.6 ± 2.0	10.6 ± 2.1	10.8 ± 1.7	0.776
IVS thickness (mm)	21.5 ± 7.1	21.5 ± 7.0	21.6 ± 7.9	0.982
LA volume (ml)	115.6 ± 56	111.7 ± 83.9	130.3 ± 87.2	0.518
LV end-diastolic volume (ml)	108.9 ± 49.7	110.8 ± 52.2	92.7 ± 7.0	0.560
LV end-systolic volume (ml)	51.6 ± 35.6	52.6 ± 37.4	44.0 ± 19.0	0.700
LV ejection fraction (%)	55.3 ± 7.8	55.6 ± 8.2	54.1 ± 6.3	0.594
LV mass (g)	301.4 ± 114.0	306.0 ± 115.2	261.0 ± 115.4	0.527
LV mass index (g/m^2^)	155.9 ± 52.3	159.0 ± 53.1	129.5 ± 43.1	0.364
Relative wall thickness	0.43 ± 0.10	0.44 ± 0.10	0.34 ± 0.02	0.103
LV outflow pressure gradient (mmHg)	8.2 ± 11.1	6.0 ± 2.5	16.1 ± 22.7	0.252
E/e′ ratio	9.7 ± 4.5	8.8 ± 3.3	13.9 ± 6.9	0.079*
TAPSE (mm)	21.4 ± 4.8	21.0 ± 4.4	22.5 ± 6.0	0.363
Systolic anterior motion, *n* (%)	10 (15.9%)	5 (10.4%)	5 (33.3%)	0.025*
Papillary muscle abnormalities, *n* (%)	4 (6.3%)	4 (8.3%)	–	0.261
Mitral leaflet abnormalities, *n* (%)	15 (23.8%)	9 (18.8%)	6 (40.0%)	0.039*
Calcification of mitral annulus, *n* (%)	3 (4.8%)	–	3 (20.0%)	0.001*

*PLW* posterolateral wall, *IVS* interventricular septum, *LA* left atrium, *LV* left ventricle, *E/e′* LV filling pressure, *TAPSE* tricuspid annular plane systolic excursion

Importantly, the systolic anterior motion was significantly higher in patients carrying MYH7 mutation (33% vs. 10%; *p* = 0.025), as well as mitral leaflet abnormalities (40% vs. 19%; *p* = 0.039). Calcifications of mitral annulus were registered only in MYH7 patients (20% vs. 0%; *p* = 0.001).

An interesting finding is the difference of E/e′ ratio—a marker of LV filling pressure—between the groups (MYBPC3 8.8 ± 3.3, MYH7 13.9 ± 6.9, *p* = 0.079). Although the level of significance is slightly beyond the threshold, the difference is indicative.

## Discussion

The genetic basis of HCM is more complex than previously thought: known genetic mutations are responsible for about half of the cases, while the remaining causes are unknown. Since variants have not been found to explain the presence of the disease in many patients, there are certainly other, yet unidentified genes. There is an emphasized need to discover additional genetic, epigenetic, and environmental causes that would explain the high proportion of cases of unknown etiology. For many newly reported genes, the lack of strong evidence to support a causal role in HCM creates uncertainty in the interpretation of the results. One of the major roles of genetic testing for HCM patients is better clinical surveillance of asymptomatic family members.

This study analyzed the genetic determinacy of various clinical phenotype parameters among patients with HCM. Only carriers of a single gene mutation, either MYBPC3 or MYH7 were included. Studies that performed genetic screening in large cohorts of patients with a confirmed clinical diagnosis of HCM managed to detect a pathogenic mutation in about 40–50% of patients [13, 15], suggesting that as much as half of the HCM diagnosed patients do not have known sarcomeric gene mutations.

The MYBPC3 and MYH7 mutations are the two most common mutations among HCM patients with identified sarcomeric gene mutations. A recent meta-analysis on 7675 HCM patients including a total of 51 studies performed by Sedaghat-Hamedani et al. [16] found that the prevalence of MYBPC3 and MYH7 gene mutations were 20% and 14%, respectively, while all the other mutations had a prevalence below 2%.

HCM is a disease of a younger age, as it is often first diagnosed before the age of 40 [15, 17]. In our study, patients’ mean age was 50 for MYBPC3 and 55 for MYH7 mutations, with no significant difference among groups. This contrasts with previous findings, which suggest earlier onset and diagnosis of the disease for MYH7 mutation [16, 18]. Patients in our study were predominantly male, which is consistent with gender distribution across literature, where about two-thirds of HCM patients are male [13, 19, 20].

Olivotto et al. [21] in their multicenter study from 2005, examined differences in HCM presentation among genders in a population of 969 patients. Although most patients were male (59%), mortality rates did not differ among genders. The authors also pointed out that female patients with HCM although more symptomatic, were under-represented and older. Females were more susceptible to advanced heart failure development, mostly due to LV outflow obstruction. Results from a more recent study from Jang et al. [22] conducted on 202 HCM patients without LV outflow obstruction are in-line with previously mentioned. Jang et al. concluded that females had a higher incidence of heart failure, as well as a greater risk of hospitalization and cardiovascular-related mortality. A higher risk of heart failure in female patients was attributed to the differences in LA and LV morphology and diastolic function between the genders.

Patients with MYBPC3 mutation in our study had a notable number (46%) of relatives with a confirmed HCM diagnosis. Across the literature, various rates of positive family history ranging from 25 to 70% have been reported [18, 19]. However, the reliability of these numbers should be taken with reserve, because family screening in patients with HCM has still not been fully implemented, despite the clear recommendations for a detailed follow-up of all adult first-degree relatives [5, 23]. New evidence suggests that screening should be performed even earlier in child age, especially in families with MYBPC3 and MYH7 mutations [24]. Moreover, the diagnosis in relatives is often established solely on phenotypic expression (i.e. imaging methods like echocardiography and cardiac magnetic resonance), without proper genetic testing. Even in the case of performed genetic analysis, currently available methods still fail to identify more than half of patients with HCM [25].

Several studies have attempted to differentiate between disease severity, progression, and phenotype-based on specific mutation subclasses, but there is currently no consensus as to whether a specific phenotype or prognosis can be predicted from an MYBPC3 mutation [26]. Mutation of the MYH7 gene is associated with an earlier onset of symptoms, more pronounced hypertrophy, and poor prognosis [27]. The Arg453Cys mutation of MYH7 is associated with a high incidence of terminal heart failure and premature death [28]. Several studies have found a correlation between five mutations (four in the MYH7 gene and one in the gene encoding cardiac troponin T) and high incidences of advanced cardiac death, however, these associations were not consistent with the results of other studies [29].

The study by Olivotto et al. [30] assessed the occurrence of atrial fibrillation and outcome in 480 consecutive HCM patients (age at diagnosis, 45 ± 20 years; 61% male) during a follow-up period of 9.1 ± 6.4 years. In their cohort, atrial fibrillation was documented in 107 patients, with a prevalence of 22%. The authors concluded that atrial fibrillation is associated with substantial risk for heart failure-related mortality, stroke, and severe functional disability, particularly in patients with outflow obstruction, those ≤ 50 years of age, or those developing chronic atrial fibrillation.

Atrial fibrillation tended to be more prevalent in the MYH7 group in our study. This finding is consistent with previous studies [17, 31], which reported a higher incidence of atrial fibrillation in patients with MYH7 mutation in comparison to other HCM patients. Since the development of atrial fibrillation was associated with risk factors such as LA enlargement, LV wall thickness, and LV outflow tract obstruction, these results suggest that patients with MYH7 mutation present with a more severe clinical phenotype. However, a prospective study on 237 HCM patients with a mean follow-up period of 14 ± 10 years found no statistically significant difference in atrial fibrillation between patients with MYBPC3 and MYH7 mutations, with an incidence of 31% and 37%, respectively [32].

Detailed analysis of echocardiography parameters between the MYBPC3 and MYH7 groups in the present study revealed a somewhat similar phenotype expression with minor differences between the groups, although with slightly more severe disease presentation in the MYH7 group. Most importantly, LV wall hypertrophy was equally expressed in both groups at the posterolateral wall and interventricular septum. Previous studies on larger groups of HCM patients that analyzed myocardial wall thickness measured by both echocardiography [16–18, 33] and cardiac magnetic resonance [20] also discovered no significant differences regarding LV wall thickness between MYBPC3 and MYH7 patients. The somewhat counterintuitive finding came from the Florence group [34], stating that LV mass index was normal in about 20% of patients with definite HCM phenotype and that increased LV mass alone should not be the parameter for establishing the clinical diagnosis of HCM. The LV mass correlated weakly with maximal wall thickness and proved more sensitive in predicting outcomes.

Heart systolic function measured through ejection fraction for LV and TAPSE for right ventricle were preserved in all study patients, with no differences between the groups. This is consistent with previous findings and the current standpoint that HCM generally does not lead to systolic function deterioration. The symptoms and clinical severity are dominantly determined by the combination of diastolic dysfunction, mitral apparatus abnormalities, and LV outflow tract obstruction [35, 36]. A recent study by Miller et al. [37] established that patients with pathogenic, likely pathogenic or rare MYH7 variants had higher LV ejection fraction than those with MYBPC3 variants (68.8 vs. 59.1, *p* < 0.001) and higher right ventricle ejection fraction (67.3 vs. 60.8, *p* = 0.018). Additionally, patients with MYBPC3 variants were more likely to have LV ejection fraction < 55% (29.7% vs. 4.9%, *p* = 0.005).

A very interesting paper from Maron et al. [38] explored mitral valve abnormalities in HCM patients using cardiovascular magnetic resonance imaging. Mitral valve morphology was observed and compared between 172 patients with HCM and 172 controls without evidence of cardiovascular disease. After careful characterization, they concluded that mitral valve abnormalities (i.e. leaflet elongation) independently contribute to the severity of HCM presentation, thus expanding the area undesirable effects of HCM genes from solely sarcomere mutations to valvular structures as well. We wanted to further classify mitral valve abnormalities depending on the genetic basis. In this regard, the MYH7 group in our study had a significantly higher number of mitral leaflet abnormalities, mitral annulus calcifications, and the most important higher number of systolic anterior motion, contributing to the worse phenotype expression of MYH7 versus MYBPC3 gene mutations. The study of Groarke et al. [39] observed an increased number of mitral valve abnormalities in patients with sarcomeric gene mutations, however, they did not analyze the difference among the particular gene mutations. Waldmuller et al. [15] on the other hand, reported a more severe level of mitral regurgitation in patients with MYH7 mutation than in patients with MYBPC3 mutation.

Diagnosis of hereditary cardiac disorders based on genetic information is particularly challenging because of the high genetic heterogeneity and overlapping and variable nature of these clinical presentations. The clinical presentation of HCM is influenced by age, lifestyle, and presence of hypertension, among other factors. Although there is still no consensus on the exact impact of gender on HCM presentation and progression, gender influence is thought to exist and that differences in gene expression and hormonal differences affect the symptoms and clinical outcomes of HCM.

Our study was able to demonstrate the subtle but clinically important difference between patients with different genetic profiles. The clinical implications that may arise from these findings point to the fact that structural abnormalities are more prevalent in MYH7 gene mutation. Patients with MYH7 mutation would probably benefit from more intense imaging surveillance that should start at a younger age as they are likely to develop mitral valve dysfunction and LVOT obstruction. Concerning diastolic dysfunction, it is reasonable to assume that patients with MYH7 gene mutation would benefit from earlier commencement and more aggressive medical treatment. Given the clinical profile, MYH7 mutation patients would be ideal candidates for cardiac myosin inhibitors such as mavacamten. Strenuous exercise should be routinely discouraged, especially in patients with the MYH7 gene mutation.

Our data also suggest and confirm already established management paradigms—an individualized approach concerning specific underlying clinical conditions and pathways (sudden cardiac death risk, heart failure, and atrial fibrillation). Such an approach has been proven to provide the opportunity to aggressively alter the progression of the disease, prevent mortality, and provide normal or extended life expectancy associated with improved quality of life.

### Study limitations

We acknowledge that the large number of operators involved in echocardiographic measurements in this multicenter study represents an unavoidable limitation. However, care was taken to standardize measurements of cardiac dimension and function by prospectively providing detailed technical instructions to all participating centers.

Finally, although the number of included patients in the study is modest, we believe that patient heterogenicity (multicenter study) confers substantial power to our data. Nevertheless, the modest size is one reason to exercise caution in extrapolating these results to the broad spectrum of hypertrophic cardiomyopathy.

A cross-sectional study design does not allow monitoring of disease progression. However, disease progression and response to pharmacological and lifestyle intervention in HCM is subject to our separate ongoing longitudinal SILICOFCM study [14, 40].

## Conclusions

Up to this point, numerous mutations leading to HCM have been identified and various clinical manifestations and phenotypic expressions of HCM have been described (from a completely asymptomatic condition, through outflow tract obstruction, diastolic dysfunction, to progressive heart failure and sudden cardiac death). However, no consistent association between the HCM genotype and phenotype have been identified.

In those terms, our study is no exception. Although we focused our attention on the two most common sarcomeric gene mutations responsible for HCM—MYBPC3 and MYH7 gene mutations—we were not able to demonstrate any substantial differences regarding clinical and echocardiography findings. More frequent systolic anterior motion and other mitral valve abnormalities as well as increased left ventricle filling pressure in MYH7 gene mutation suggests that MYH7 gene mutation does present with a more severe disease phenotype.

Correlation between the genetic and clinical status of HCM patients remains elusive in most of the cases—limitation with a major impact on the development of personalized medicine approaches. Our study might subtly add to the overall understanding of such complex relations and might push genetic testing results from strictly diagnostic to prognostic fashion.

## Data Availability

The datasets used and analyzed during the current study are available from the corresponding author on reasonable request.

## Abbreviations

ACTC: α-Actin
BMI: Body mass index
BSA: Body surface area
CO: Cardiac output
E/e′: Left ventricle filling pressure
HCM: Hypertrophic cardiomyopathy
HR: Heart rate
IVS: Interventricular septum
LA: Left atrium
LV: Left ventricle
LVEDV: Left ventricular end-diastolic volume
LVESV: Left ventricle end-systolic volume
LVmass: Left ventricle myocardial mass
MYBPC3: Myosin-binding protein C
MYH7: β-Myosin heavy chain
PLW: Posterolateral wall
RWT: Relative wall thickness
SV: Stroke volume
TAPSE: Tricuspid annular plane systolic excursion
TNNI3: Troponin I type 3
TNNT2: Troponin T type 2
TPM1: α-Tropomyosin
